# Pet rodents as possible risk for leptospirosis, Belgium and France, 2009 to 2016

**DOI:** 10.2807/1560-7917.ES.2017.22.43.16-00792

**Published:** 2017-10-26

**Authors:** Marcella Mori, Pascale Bourhy, Marine Le Guyader, Marjan Van Esbroeck, Zorée Djelouadji, Alexandra Septfons, Angeli Kodjo, Mathieu Picardeau

**Affiliations:** 1 Veterinary and Agrochemical Research Center, CODA- CERVA, Unit “Bacterial Zoonoses of Livestock”, Operational Direction Bacterial Diseases, Brussels, Belgium; 2 Institut Pasteur, Unité Biologie des spirochètes, CNR de la Leptospirose, Paris, France; 3 Campus Vétérinaire - VetAgro Sup, Laboratoire des Leptospires, Marcy l'Etoile, France; 4 Institute for Tropical Medecine, Department of Clinical Sciences, Antwerp, Belgium; 5 Santé publique France, French national public health agency, Saint-Maurice, France

**Keywords:** zoonosis, leptospirosis, pet rodent

## Abstract

Leptospirosis is an under-reported and emerging zoonotic disease which is potentially fatal in humans. Rodents are the main reservoirs for pathogenic *Leptospira* spp., but diagnosis in these animals is difficult, and their infection, which does not induce symptoms, usually goes unoticed. Although the exposures of most human cases of leptospirosis are poorly documented, we were able to identify six human cases of leptospirosis which were associated with direct contact with pet rodents (mice or rats) in Belgium and France between 2009 and 2016. All cases had severe disease and for all, the presence of *Leptospira* spp. DNA in the kidneys of their pet animals was confirmed, strongly suggesting that excretion of leptospires in urine was the way of transmission. Half of the cases shared the serogroup Icterohaemorrhagiae, which is usually associated with severe disease, with the pet rats which they were in contact with. With the popularity of rats and mice as pets, this study should contribute to raising awareness on asymptomatic pet rodents as a source of *Leptospira* infections.

## Introduction

Leptospirosis is an important zoonosis, as all mammals, including marine and flying mammals such as pinnepeds and bats, respectively, can carry pathogenic *Leptospira* species [1,2]. Studies also reported that birds, reptiles, amphibians, and fish [3,4] may act as reservoir.

In a landmark study from Inada et al. in 1916, the role of rats as a major reservoir for transmission to humans was already described [5]. Rodents, including rats and mice, are an asymptomatic reservoir, but infections in other animals can cause a wide range of manifestations including abortion, acute kidney injury, liver failure, loss of milk production, pulmonary haemorrhage syndrome, uveitis, and weight loss [2]. Pathogenic *Leptospira* colonise the proximal renal tubules of reservoir hosts, from where they are excreted via urine into the environment where they can survive in water for several months in favourable conditions [6].

Humans are considered incidental hosts and infection occurs predominantly by contact of abraded skin or mucous membranes with water or moist soil contaminated with urine of infected animals [7]. Pathogenic leptospires then disseminate haematogenously to cause a wide spectrum of clinical symptoms ranging from mild fever, to icteric Weil’s disease and pulmonary haemorrhage syndrome with possible fatal outcomes [7]. Leptospirosis is estimated to cause more than 1 million severe cases and ca 60,000 deaths per year worldwide [8]. The number of reported cases in Europe is increasing since 2014 [9] and this is probably due to climate change [10,11].

The epidemiology of leptospirosis in industrialised countries has changed over the preceding decades. The traditional patterns of occupational exposure by direct contact with infected animals (farmers, veterinarians, abattoir workers) or indirect contact (sewer workers, miners, soldiers, fish farmers, etc.) have become less frequent. By contrast, there is a considerable risk associated with recreational exposures ocurring in freshwater sports (canoeing, kayaking, rafting, and triathlons) [12-16]. Contact with pet rats has also been described as an emerging risk factor associated with leptospirosis in Germany [17]. However, few cases of potential transmission from pet rodents to humans have been published so far [18-21]. To address this lack of information, the purpose of this study is to describe clinical cases with strong diagnostic and epidemiological evidence of pet rodents as the source of infection.

## Methods

### Epidemiological investigation

When this study was conducted, reporting of leptospirosis was mandatory in the Wallonia region but not Flanders and Brussels regions of Belgium or in France. In both countries, when leptospirosis is suspected, patient serum samples are usually sent from hospitals to a number of laboratories for confirmation, together with information on possible exposures. For this retrospective study, cases of leptospirosis were found by searching records of different laboratories that perform leptospirosis diagnosis in Belgium and France, such as, the Institute for Tropical Medecine (Antwerp, Belgium), CODA-CERVA (Brussels, Belgium), the National Reference Center for Leptospirosis (NRC) at the Institut Pasteur (Paris, France), and La croix Rousse Hospital (Lyon, France). Cases were defined as patients with symptoms consistent with leptospirosis (most common symptoms: fever > 39 °C, asthenia, headache, myalgia, nausea, and diarrhoea) and confirmatory laboratory results by enzyme-linked immunosorbent assay (ELISA) IgM, microscopic agglutination test (MAT) or quantitative (q)PCR. Following diagnosis, cases had been queried regarding their exposures in the month before symptom onset (e.g. farming, gardening, water exposure, travel in endemic countries, professionals activities) and their contact with animals. When contact with pet rodents was reported, the pet rodents were euthanised after obtaining the approval from the owner, and blood, pleural fluid (only for pets of cases 2, 3, and 4) and kidney samples were sent to reference laboratories. Informed consent was obtained from cases on the possible use of their clinical and laboratory information for scientific purpose. 

### Diagnosis of human leptospirosis

Diagnosis was performed at the National Reference Center for Leptospirosis (NRC) at the Institut Pasteur (Paris, France), La croix Rousse Hospital (Lyon, France), the Institute for Tropical Medecine (Antwerp, Belgium), and CODA-CERVA (Brussels, Belgium). Serum samples from patients were tested by an ‘in house’ ELISA IgM [22] and/or an immunochromatographic *L. biflexa* Patoc antigen-based assay (Core diagnostics, Birmingham, United Kingdom) in combination with MAT. MAT was conducted using the following antigens: serogroups Australis (serovar Australis), Autumnalis (serovar Autumnalis), Ballum (serovar Castellonis), Bataviae (serovar Bataviae), Canicola (serovar Canicola), Cynopteri (serovar Cynopteri), Grippotyphosa (serovar Grippotyphosa), Sejroe (serovars Hardjo and Sejroe), Hebdomadis (serovar Hebdomadis), Icterohaemorrhagiae (serovar Copenhageni), Panama (serovar Panama), Pomona (serovar Pomona), Pyrogenes (serovar Pyrogenes), and Tarassovi (serovar Tarassovi). MAT with a titre > 1:100 was considered as positive. The highest agglutination titre of the serum with one particular pathogenic *Leptospira* was taken to identify the presumptive serogroup of the infecting bacterium.

### Typing of *Leptospira* strains in pet rodents

Isolation was attempted by inoculating urine or kidney extracts of pet rodents into EMJH liquid medium. *Leptospira* positive cultures were then typed by molecular methods and serogrouping using MAT. Typing of animal samples was performed at the NRC at the Institut Pasteur (Paris, France), CODA-CERVA (Brussels, Belgium), and Laboratoire des Leptospires (Marcy l’Etoile, France). DNA was extracted from rodent kidneys (QIAamp DNA Mini Kit; Qiagen SA, and MagMAX Technology protocol). Species identification was performed by amplification and sequencing of the 16S rRNA [23]. Identification at the subspecies level was performed by multilocus variable-number tandem repeat analysis (MLVA) using the loci VNTR4, VNTR7, and VNTR10 [24]. MAT was also performed on pleural fluids or sera of euthanised animals of cases 2, 3 and 4, respectively.

## Results

### Finding of human leptospirosis cases

During the years 2009 to 2016, we identified six leptospirosis cases for which pet rodents were the source of infection in Belgium and France. The median age was 31 years (21–57) and four cases were women. All cases were hospitalised because of severe disease. On admission, four patients had elevated liver enzyme or bilirubin levels, C-reactive protein was high in four patients, two were thrombocytopenic, one had hepatic cytolysis, and one had meningitis/meningoencephalitis. Patients did not report any specific medical history nor immunosupression. Beta-lactam or cephalosporin antibiotics were prescribed for all patients. One of the cases required neurological follow-up for a concentration disorder.

Based on the results of MAT, the presumptive causal agent of the infection was identified as serogroup Icterohaemorrhagiae in three cases and serogroup Sejroe in one case. Sera from the two remaining cases were positive by ELISA IgM but did not demonstrate specific agglutination with any pathogenic *Leptospira* tested in acute sera by MAT (Table). Because culture of acute blood samples and urine was not performed, we were not able to identify the infecting serovar.

**Table:** Characteristics of human cases of leptospirosis with direct contact with pet rodents, Belgium and France, 2009–2016 (n = 6 cases)

Case number	Sex	Age in years	Date of symptom onset	Country, province/ region	Number of days after symptom onset when serum was sampled	Laboratory results		Infected pet (number of animals)	Origin of the pet(s)	Species/ serogroup^a^ in pet
						ELISA IgM	MAT			
1	F	44	May 2009	France, Auvergne-Rhône-Alpes	1	Pos	Sg Sejroe	Mouse (3)	Pet shop	*L. borgpetersenii* sg Sejroe
2^b^	M	23	Jun 2013	Belgium, East Flanders	1	Pos	Sg Semaranga^c^	Mouse (1)	Pet shop	Sg Ballum or Pyrogenes
					3	Pos	Sg Semaranga^c^			
3^b^	F	21	Aug 2013	Belgium, East Flanders	1	Pos	Neg			
4	F	22	Nov 2013	Belgium, East Flanders	1	Neg	Neg	Rat (7)	Pet shop and exchange within a ‘pet-rat’ community	*L. interrogans* sg Icterohaemorrhagiae
					7	Pos	Sg Icterohaemorrhagiae			
5	M	21	Feb 2015	France, Lorraine	15	Pos	Sg Icterohaemorrhagiae	Rat (1)	Pet shop	*L. interrogans* sg Icterohaemorrhagiae
6	F	57	Jul 2016	France, Provence-Alpes-Côte d’Azur	18	Pos	Sg Icterohaemorrhagiae	Rat (1)	Pet shop	*L. interrogans* sg Icterohaemorrhagiae
					40	Pos	Sg Icterohaemorrhagiae			

ELISA: enzyme-linked immunosorbent assay; MAT: microscopic agglutination test; neg: negative; pos: positive; sg: serogroup.

^a^ Serogroup was determined by MAT on pleural fluids or sera of cases 2, 3 and 4, respectively, or typing of strains isolated from pets (cases 1, 4, 5, and 6). For serum samples from mouse of cases 2 and 3, the MAT titres could not differentiate between serogroups Ballum and Pyrogenes and the *Leptospira* species could not be determined either.

^b^ Cases 2 and 3 were living in the same apartment and exposed to the same pet rodent.

^c^ Serovar Patoc from the serogroup Semaranga is a non-pathogenic *Leptospira* strain, however agglutination with this serovar together with severe disease can be indicative of an infection with a pathogenic serogroup.

The epidemiological investigation revealed that one patient (case 1) was a magician who used mice for magic tricks in nurseries. Cases 2 and 3 were living in the same apartment and owned a pet mouse. Case 4 worked in a pet shop, was the owner of eleven pet rats, and was a volunteer in a pet-rat daycare association. Cases 5 and 6 were keeping pet rats from a friend or daughter, respectively. For all the cases, besides the exposure to the pet rats, no other specific exposures were identified. Pets had been purchased from pet shops in Belgium and France (Table). All patients were regularly exposed to their pet rodents which were usually kept in cages and occasionally left to freely roam in the household. No particular preventive measures were taken by any of the patients when caring and handling the animals.

### *Leptospira* spp. infections in pet rodents

All pet rodents were euthanised a few months after the diagnosis of leptospirosis in the patients living with the pets. MAT was performed on pleural fluid or serum samples of euthanised animals of cases 2, 3 and 4, whereby the serogroup was inconclusive for the mouse of cases 2 and 3 and Icterohaemorrhagieae for the rat of case 4 (Table). Urine and kidney of pet rats and mice were tested for *Leptospira* by PCR analysis and bacterial culture. All kidneys proved to be positive by PCR. Sequencing of the *Leptospira* 16S rDNA gene indicated that the pets from cases 4, 5, and 6 were infected with *L. interrogans* and the pet from case 1 with *L. borgpetersenii*. In addition, cultures were positive for *Leptospira* in the pets from cases 1, 4 and 5. Serogrouping of isolates with rabbit antisera against reference serovars showed strong agglutination with the serogroups Icterohaemorrhagiae and Sejroe (Table). Subsequently antiserum against the Sejroe isolate from case 1 with reference serovars from the serogroup Sejroe revealed that the highest agglutination titre was against the serovar Saxkoebing. MLVA, which is a PCR-based method for the identification of most of the serovars of *L. interrogans* and *L. kirschneri* [24], of DNA extract from kidneys or isolates confirmed the presence of serovars Icterohaemorrhagiae or Copenhageni (both from the serogroup Icterohaemorrhagiae) DNA in the positive rats from cases 4, 5, and 6.

Except for cases 2 and 3, the infecting strain in pets was in agreement with the serogroups determined by MAT in the corresponding patients, further confirming that the pet rodents were responsible for the transmission of the disease (Table).

## Discussion

Over the last decade, ‘exotic’ pet mammals species, such as ferrets, rabbits, and rodents, which are known to carry zoonotic pathogens such as parasites, viruses and bacteria, have become increasingly popular [25]. A study between 2011 and 2013 on leptospirosis in free-living rodents in an urban setting in France showed 43% of rats infected [26]. In Germany in 2007, seven of 11 common voles tested on a farm during a human outbreak investigation were positive for *Leptospira* [27]. Pet rodents likely become infected with *Leptospira* spp. by exposure to wild infected animals, or by environmental contact with the bacteria [28]. In a study conducted between 2008 and 2009, pet rat owners were identified to be at risk for exposure to pathogenic leptospires [17]. Companion rodent pets living in close proximity to humans can therefore be a cause of transmission of leptospirosis. 

In Europe, the incidence of leptospirosis is lower than 1 per 100,000 inhabitants, with France reporting a relatively high incidence (1/100,000 inhabitants in 2014 and 2015) [9,12,29,30]. In this study, we identified six cases in France and Belgium with a history of contact with pet rodents. For four of six cases, the infecting strain was in agreement with the serogroup and genotype found in the corresponding pet rodents, consistent with the pets being responsible for the transmission of the disease. This study confirms previous reports [18-21], indicating that client-owned pet rodents may serve as carriers for pathogenic *Leptospira* spp. At least three cases were due to serogroup Icterohaemorrhagiae which is most often implicated with severe infections and fatal outcomes [31-33]. Moreover, post-acute infection neurological sequelae were reported by one patient. The presence of such sequelae, which can last up to 24 months [34], is relevant when assessing the public health burden of leptospirosis

Environmental transmission has frequently been considered the major route of *Leptospira* infection among rodents. However, the role of breastfeeding and sexual and *in utero* transmissions in rodents remains to be determined. A recent study identified leptospires in the milk and breast tissue of wild rats, suggesting the possibility of milk-borne transmission of *Leptospira* to neonates [35]. The presence of unrecognised *Leptospira* infections in animal care facilities may be due to accidental introduction of *Leptospira* into rodent colonies by interactions with wild rodents. The exchange of rodents among husbandries or pet shops can also be a source of sporadic *Leptospira* infections. 

Currently, rodent colonies in pet shops are not screened for *Leptospira* infection or any other zoonotic diseases. Surveillance is based upon veterinary check-up of the clinical status of the animal. Asymptomatic pet rodents carrying pathogenic *Leptospira* spp. will not be detected by this type of screening. Since serology or PCR in urine samples may not be conclusive for the diagnosis of leptospirosis, screening of rodents can only be performed by qPCR on kidneys after euthanasia of the animals. Because quarantine and antibiotic treatment of infected animals may not be effective in eliminating the *Leptospira* infection, in case of detection of infected rodents, euthanasia of the whole colony seems to be the only method of control. Ideally, pet rodents would come from highly controlled breeding centres (with a ‘free of the disease’ status) and the handling and management of animals would follow specific preventive guidelines to minimise the introduction and potential transmission of *Leptospira* infections. Since skin abrasions serve as portals of entry for the leptospires, pet owners or people living in close proximity of pet rodents should use gloves, or at least protect skin cuts or abrasions with bandages, when handling rodents or cleaning cages to prevent transmission. Specific information about transmission of the bacteria and preventive measures should be provided to the pet owners.

In conclusion, this study emphasises the potential of pet rodents to be infected with *Leptospira*. We recommend that the health surveillance schemes of rodents in animal care facilities target pathogenic *Leptospira* spp. New human cases of leptospirosis should be carefully documented in order to identify contact with pet rodents as the source of infection. People purchasing pet rodents or living/working in close contact with such animals should be informed about the risk of contracting leptospirosis and on preventive measures to reduce this risk. 
